# Rpt5-Derived Analogs Stimulate Human Proteasome Activity in Cells and Degrade Proteins Forming Toxic Aggregates in Age-Related Diseases

**DOI:** 10.3390/ijms25094663

**Published:** 2024-04-25

**Authors:** Katarzyna Cekała, Karolina Trepczyk, Julia Witkowska, Elżbieta Jankowska, Ewa Wieczerzak

**Affiliations:** Faculty of Chemistry, University of Gdańsk, Wita Stwosza 63, 80-308 Gdańsk, Poland; katarzyna.jedrzejewska@phdstud.ug.edu.pl (K.C.);

**Keywords:** proteasome, activator, aging, age-related diseases

## Abstract

Aging and age-related diseases are associated with a decline in the capacity of protein turnover. Intrinsically disordered proteins, as well as proteins misfolded and oxidatively damaged, prone to aggregation, are preferentially digested by the ubiquitin-independent proteasome system (UIPS), a major component of which is the 20S proteasome. Therefore, boosting 20S activity constitutes a promising strategy to counteract a decrease in total proteasome activity during aging. One way to enhance the proteolytic removal of unwanted proteins appears to be the use of peptide-based activators of the 20S. In this study, we synthesized a series of peptides and peptidomimetics based on the C-terminus of the Rpt5 subunit of the 19S regulatory particle. Some of them efficiently stimulated human 20S proteasome activity. The attachment of the cell-penetrating peptide TAT allowed them to penetrate the cell membrane and stimulate proteasome activity in HEK293T cells, which was demonstrated using a cell-permeable substrate of the proteasome, TAS3. Furthermore, the best activator enhanced the degradation of aggregation-prone α-synuclein and Tau-441. The obtained compounds may therefore have the potential to compensate for the unbalanced proteostasis found in aging and age-related diseases.

## 1. Introduction

One of the hallmarks of aging is impaired proteostasis. The activities of the two main protein clearance systems, namely, the autophagy–lysosomal system and the ubiquitin–proteasome system (UPS), decline with age [1,2]. Consequently, damaged proteins that are efficiently cleared in young cells tend to accumulate with age, impairing cell function. Accordingly, the prevalence of diseases considered age-related, such as Alzheimer’s (AD), Parkinson’s (PD), or Huntington’s disease (HD), increases dramatically with age [3].

An essential component of the ubiquitin–proteasome system, responsible for controlling the proteolysis, is the 20S proteasome (core particle, CP) [4,5]. The eukaryotic CP is a barrel-shaped structure composed of four stacked heptameric rings arranged in the αββα fashion with a central pore providing space for substrate entry [6]. The inner β-rings contain three catalytic subunits—β1, β2, and β5—which have unique proteolytic activities (caspase-like (C-L), trypsin-like (T-L), and chymotrypsin-like (ChT-L), respectively) and are responsible for cutting the linear protein sequence into short peptides [7]. The substrate entry is regulated by the gate mainly formed by the *N*-termini of α2, α3, and α4 subunits [8]. The opening of the gate can be triggered through the binding of proteasome activators (PAs), which determine whether 20S is coupled to the ubiquitin–proteasome system or ubiquitin-independent proteasome system. The canonical regulatory particle of the UPS is the 19S (PA700, regulatory particle (RP)), which, together with the 20S, composes the 26S complex degrading ubiquitinated proteins. The 19S is composed of two subcomplexes: a base and a lid. A lid consists of nine non-ATPase subunits, while a base is formed with four non-ATPase subunits and a heterohexameric ring of ATPases (Rpt1–6), three of which are terminated with a conserved tripeptide sequence, the HbYX (hydrophobic-penultimate Tyr-any amino acid) [9]. To allosterically stimulate gate-opening the 19S docks the C-terminal tails of Rpt subunits in the intersubunit pockets of the 20S α ring.

The major player in the UIPS system is the 20S proteasome itself, of which its activity may be amplified by specific PAs, including PA200 and PA28/11S [10,11]. The 20S cleaves proteins that are capable of entering into its catalytic chamber without previous unfolding. These substrates include intrinsically disordered proteins (IDPs) as well as proteins containing intrinsically disordered regions (IDRs), and those that have lost their native structure due to mutations, oxidative damage and aging. As we age, reactive oxygen species from exogenous sources and cellular metabolism accumulate what results in significant damage in proteins, making them prone to aggregation [12]. Although oxidative stress is accompanied by the disassembly of the 26S proteasome into its 20S and 19S components, an increase in the content of the 20S core molecule enables cells to efficiently remove oxidatively damaged proteins [13,14,15].

About 30% of cellular 20S proteasomes are associated with 19S, and more than 50% are found in a free form. The remaining 20% are bound to UIPS-specific PAs, PA28, and PA200 [16]. The 20S:26S ratio has been reported to increase with age. In aged cells, it is estimated that about 66% of proteasomes exist as free 20S, and less than 10% is bound to PAs specific to UIPS while 21–35% of the total proteasome pool is 26S [17]. A decreased 26S level is a key component of the decline in total proteasome activity in many cells, tissues, and organisms during aging [18]. However, the large pool of free 20S along with the fact that proteins that accumulate in aging and age-related diseases are preferentially degraded by UIPS, makes the 20S an attractive therapeutic target that can be activated to compensate for reduced proteasomal function [19].

One way to enhance the proteolytic removal of unwanted proteins appears to be the use of small-molecule activators of the 20S. Several small-molecule proteasome enhancers have been described in the last few years. Among them, chlorpromazine [20] and imidazoline TCH-165 [21], in vitro, enhanced 20S proteasome activity and promoted the degradation of α-synuclein and tau, of which their aggregates have been observed in Parkinson’s and Alzheimer’s disease, respectively. Two other compounds, AM-404 and MK-886 have been shown to enhance the digestion of α-synuclein in human embryonic kidney 293T (HEK293T) cells [22]. Moreover, Liao et al., by evaluating green fluorescent protein (GFP) cleavage from a tau-GFP fusion protein expressed in HEK293T cells, confirmed that MK-886 stimulates proteasome in cellulo [23].

Peptides and peptide-based compounds that usually possess higher potency and selectivity than small molecules, represent another class of proteasome activators. Synthetic proteasome-activating peptide 1 (PAP-1) increased the ChT-L activity of the 20S and prevented the aggregation of superoxide dismutase 1 (SOD1) in a cellular model of amyotrophic lateral sclerosis [24]. Peptide-based proteasome agonists can also be derived from the binding regions of the natural proteasome regulators containing the HbYX motif. We have recently showed that a 14-residue peptide, Blm-pep, whose sequence was derived from the Blm10 activator, efficiently stimulates all three peptidases of human 20S proteasome [25]. Moreover, peptidomimetics based on the sequence of Blm-pep were able to enhance the 20S-mediated degradation of natively unfolded and oxidized protein substrates, including α-synuclein [26]. Proline- and arginine-rich (PR) peptides modified with C-terminal HbYX residues efficiently degrade α-synuclein and activate proteasome in cultured fibroblasts [27]. Recently, cyclic peptides that stimulate the 20S were described. These compounds selectively degraded α-synuclein and efficiently stimulated the proteasome in HEK293T cells [28].

In the current work, we synthesized a series of peptides and peptidomimetics based on the binding sequence of the natural proteasome activator, 19S, and demonstrated that several of them efficiently stimulate human 20S (h20S) activity. After the attachment of the cell-penetrating peptide TAT, the compounds were able to penetrate the cell membrane and stimulate proteasome activity in HEK293T cells. In vitro protein degradation assays revealed that the most potent activator TAT-30 stimulates the proteasomal degradation of α-synuclein and Tau-441, which form toxic aggregates in age-related Parkinson’s and Alzheimer’s diseases.

## 2. Results and Discussion

**Figure 1:**
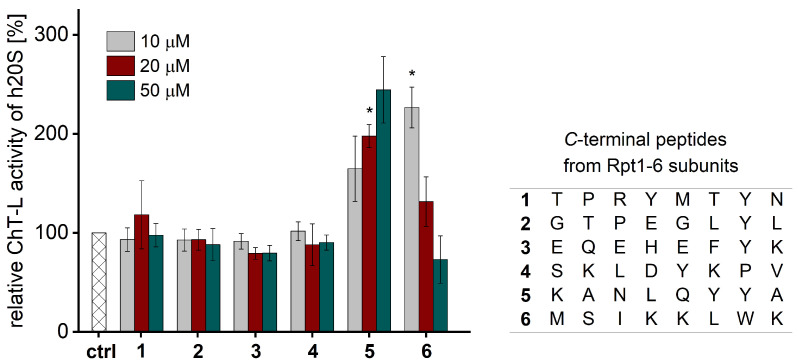
The capacity of compounds **1**–**6** for stimulating ChT-L activity of human 20S proteasome. Only the peptide corresponding to the C-terminus of the Rpt5 subunit (**5**) increased the activity of the 20S in a dose-dependent manner. All activity assays were performed in three independent replicates. Results are expressed as a percentage of activity of the latent 20S and are presented as the mean ± standard deviation (SD). Ordinary one-way ANOVA was used to determine statistical significance (* *p* < 0.05).

In the search for compounds able to stimulate 20S proteasome activity in human cells, we focused our attention on the binding region of the natural proteasome regulator, 19S. Peptides corresponding to the C-terminus of the Rpt2 and Rpt5 subunits of the 19S, which contain the HbYX motif, were able to induce the hydrolysis of short peptide substrates through rabbit [29] or bovine 20S proteasome [30]. In our work, we aimed to investigate the influence of the peptides derived from the C-terminal fragments of Rpt1-Rpt6 subunits on the human 20S proteasome. A study by Smith et al. [29] showed that only peptides with seven residues or longer could stimulate the activity of rabbit muscle 20S proteasome. Recently, it was also described that the 6- and 7-amino-acid-long peptides derived from the Rpt5’s C-terminus were significantly more potent stimulators of human 20S than shorter ones [31]. Therefore, we synthesized 8-amino acid peptides (compounds **1**–**6**, Figure 1) and tested their effect on the proteolytic activity of the human 20S using small fluorogenic peptide substrates that contain a 7-amino-4-methylcoumarin reporter group (AMC) and probed the activity of the catalytic sites in the 20S: chymotrypsin-like (Suc-LLVY-AMC), trypsin-like (Boc-LRR-AMC), and caspase-like (Z-LLE-AMC). The obtained compounds were compared based on their ability to stimulate the hydrolysis rate. Since ChT-L is considered a rate-limiting “workhorse” and is the major target for proteasome modulators, we focused our attention on this peptidase.

The activity assays demonstrated that only the C-terminal peptide from the Rpt5 subunit (**5**) stimulated the ChT-L, T-L, and C-L peptidases of the human 20S (Figure 1 and Figure S1). At a concentration of 50 µM, the activity of each catalytic site increased about three times. Interestingly, peptide **6**, lacking the HbYX motif, did not activate the ChT-L peptidase over the entire concentration range. At lower concentrations, the peptide stimulated 20S activity in a dose-dependent manner with maximum activation observed at 5 μM, followed by a systematic decrease in efficacy (Figure S2). This unusual bell-shaped dose response may be explained by the presence of a low-affinity secondary binding site that exerts an inhibitory effect on the ChT-L activity. A similar effect was noticed previously for the proline- and arginine-rich peptides [27] and for the short peptides derived from HIV-1 Tat protein [32]. The observed gain in the substrate turnover as a result of the activity of compound **5** is in agreement with the findings described for the stimulation of rabbit [29] and bovine 20S proteasome [30]. The ChT-L activity of these two mentioned mammalian proteasomes was also stimulated by an 8-amino acid peptide from the C-terminus of the Rpt2 subunit, which was not observed in the case of the human 20S (Figure 1). However, the concentrations of the peptides used in the above studies were much higher (250 µM for the rabbit and 400 µM for the bovine proteasome). Moreover, even at this high concentration, the Rpt2-derived peptide stimulated the bovine proteasome to a much lesser extent than the peptide corresponding to the C-terminus of Rpt5 [30].

**Figure 2:**
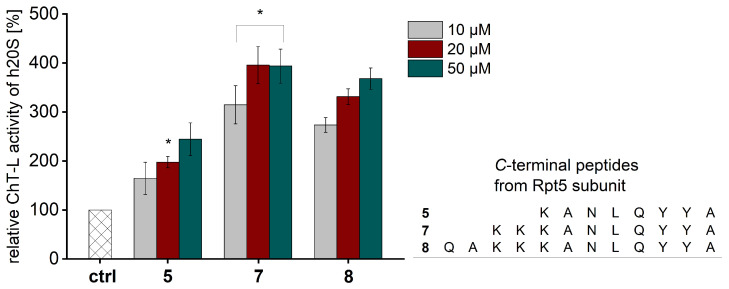
The influence of the peptide chain length on the ChT-L activity of the human 20S proteasome. Extending the length of the modulator from 8 to 10 amino acids resulted in a noticeable increase in the stimulating capacity of ChT-L peptidase, while the further elongation of the peptide chain to 12 amino acids did not cause gain in the activity of the 20S. Results are expressed as a percentage of the activity of the latent 20S and are presented as the mean ± SD. Ordinary one-way ANOVA was used to determine statistical significance (* *p* < 0.05).

In the next step of our study, we decided to check if the length of the peptidic stimulator derived from the Rpt5 subunit of 19S influenced its activating propensity toward the h20S proteasome. Therefore, we synthesized 10- and 12-residue peptides and tested their stimulation capacities for the 20S. We found that extending the peptide chain length of the modulator to 10 amino acids (compound **7**) increased its influence on all three 20S peptidases, while the further extension of the chain did not significantly affect the 20S activities (Figure 2 and Figure S3).

**Figure 3:**
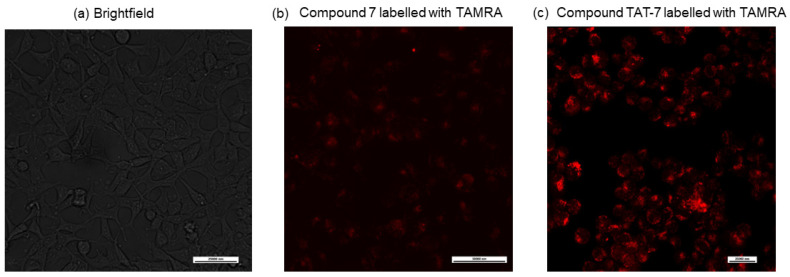
Confocal microscopy images used to illustrate the cellular internalization of compounds **7** (**b**) and TAT-7 (**c**) labeled with a fluorescent dye, TAMRA. These images demonstrate that the C-terminal peptide from the Rpt5 subunit (**7**) penetrated the cell membrane of HEK293T cells only to a small extent in contrast to its counterpart with the TAT peptide. Brightfield image of HEK293T cells is shown in (**a**). The scale bar is 25,000 nm for (**a**) and (**c**), and 50,000 nm for (**b**), respectively.

One of the obstacles in the development of peptide drugs is their low cell permeability. To evaluate if the peptide-based proteasome activators can cross the cell membrane, we synthesized an analog of peptide **7**; labeled the ε-amine group of the N-terminal lysine residue with a fluorescent dye, 5/6-carboxytetramethylrhodamine (TAMRA); and used confocal microscopy to detect its entry into cells. Figure 3b shows that the compound is only slightly cell permeable. Therefore, **7** was combined with the HIV-1 TAT protein-derived, membrane-penetrating peptide sequence GRKKRRQRRR (TAT 48–57) [33], separated by the 20S proteasome recognition sequence, LLVY. The obtained peptide (compound TAT-7) was labeled with TAMRA and its cell permeability was evaluated via confocal microscopy (Figure 3c), which indicated that it crossed the cell membrane.

The increased activity of the human 20S after treatment with peptide **7**, as well as the cell permeability of its analog linked to the TAT, prompted us to carry out structure–activity relationship studies on this Rpt5-derived compound to obtain more efficient stimulators of the 20S proteasome activity. The peptidic 20S activators with structures that are based on the natural PAs carry the HbYX motif that is necessary to associate with the proteasome. The crucial role of this tripeptide was recently confirmed by Chuah et al. [34], who designed a small molecule, ZYA, functionally mimicking the HbYX motif, that was able to activate bovine 20S proteasome. Nevertheless, the identities of residues adjacent to HbYX could have an appreciable influence on proteasome activation. To elucidate the importance of the individual residues upstream of the HbYX motif in compound **7**, we designed a series of peptides, varying the positions 1, 2, 3, 4, 5, 6, and 7. We obtained compounds with these positions containing basic, acidic, or neutral residues.

**Figure 4:**
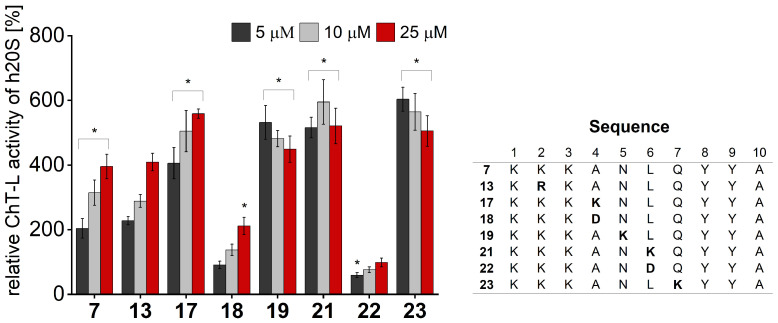
ChT-L peptidase of human 20S proteasome was activated in a dose-dependent manner by most analogs of **7**, with the exception of **22**. Compound **17** at a 25 µM concentration caused a 1.4-fold gain in activity relative to **7**. All activity assays were performed in three independent replicates. Results are expressed as a percentage of the activity of the latent 20S and are presented as the mean ± SD. Ordinary one-way ANOVA was used to determine statistical significance (* *p* < 0.05).

We performed activity assays for the obtained compounds and compared their stimulating influence on the 20S activity with the parent peptide **7** (Figure 4, Figures S4 and S5). The results of our studies indicate that the modification of position 1 with negatively charged or uncharged side chains had a modest effect on the stimulating capacity. Basic residue seemed to be required at position 2, since neither acidic (Glu) nor neutral residue (Gln) was tolerated. This fact was further confirmed by the activating propensities of peptide **13**, which in the studied position had Arg residue. As opposed to that, we found that the requirements for position 3 are more permissive. Introducing either Gln or Glu did not significantly alter the capacity of the obtained analogs to stimulate the 20S activities. Modifications at position 4 had the greatest impact on the rate of hydrolysis. The incorporation of the acidic residue remarkably ablated the activating capacity of peptide **18**, while Asn and Lys caused gain in activity. The greatest stimulatory effect was observed in the case of peptide **17**, which, at a 25 µM concentration, exhibited a 1.4-fold increase in the ChT-L and a 2.5-fold increase in the C-L activity of the 20S relative to the parent compound **7** (Figure 4 and Figure S4). We found that proteasome had sequence preferences at positions 5, 6, and 7, with Lys favored over the other residues that were tested. Introducing Lys at each of the above-mentioned positions caused a 1.1–1.3-fold gain in the ChT-L peptidase activity. An acidic residue was tolerated at position 5 and position 7 (compounds **20** and **24**, Figure S5), while the substitution of Leu with Asp at position 6 completely ablated the capacity of analog **22** for stimulating human 20S proteasome.

It is tempting to describe the h20S activation caused by peptide activators, such as that presented here with Rpt analogs or our developed Blm-pep [25], as being a result of gate opening. Both Blm-pep and the C-terminal fragment of the Rpt5 subunit contain the HbYX motif, which in natural proteasome activators, Blm10, and 19S, docks into pockets between adjacent α-subunits of the 20S, causing gate opening. Blm-pep and Rpt5-derived peptides efficiently stimulate human 20S proteasome activity; therefore, they most probably also induce the opening of the channel through which substrates enter the 20S particle. However, precise structural information on the mechanism of action of peptide proteasome activators is lacking. The crystal structure of Blm-pep bound to yeast proteasome is not helpful since the peptide was unable to stimulate the yeast enzyme, and this may be a reason why only an intermediate but still closed conformation was observed [25]. The 7–8 residue C-terminal peptides of Rpt2 and Rpt5 subunits have been shown to efficiently stimulate the bovine and rabbit proteasome in vitro [29,30], but although the authors claimed that this occurred through an allosteric conformational change that opened the gate, they did not provide any structural proof. Recently, Opoku-Nsiah et al. [31] attempted to determine via cryo-EM the structure of human 20S proteasome bound to a peptide NLSYYT, which contains the HbYX motif, but they were unable to obtain high-resolution structures. Therefore, they genetically installed this sequence at the C-termini of the activation loop-free mutant of the archaeal PA26 activator, which allowed them to determine interactions that promote gate opening in the human 20S proteasome. However, the relevance of interactions provided through this multivalent construct to the mechanism of activation induced by a peptide containing only a single HbYX motif still needs to be clarified.

In parallel, we decided to verify which peptide bonds are preferentially cleaved by the proteasome. Parent peptide **7** was incubated with the latent human 20S proteasome in Tris-HCl buffer for 3 h. After this time, the reaction was stopped, and the products were analyzed via LC-MS. The detected *m*/*z* signals, corresponding to the individual fragments of the compound, allowed the identification of cleavage sites at positions 3, 4, 8, and 9 (Figure S6). Therefore, we introduced, in the indicated positions, unnatural amino acids to improve the stability of the modulators to the 20S while maintaining their stimulating properties. At position 3, homoarginine and the uncharged isostere of Arg, citrulline, were placed. Aminobutyric acid and beta-alanine replaced naturally occurring alanine at position 4. Hydrophobic noncanonical norleucine was introduced in place of tyrosine 8 in the conserved hydrophobic-tyrosine-any amino acid (HbYX) motif. We did not exchange tyrosine in position 9 since this residue in the HbYX motif is necessary for maintaining the stimulating capacity of the 20S modulators [30,35]. We also synthesized the peptidomimetic with a reduced peptide bond between Ala and Asn residues (compound **29**).

**Figure 5:**
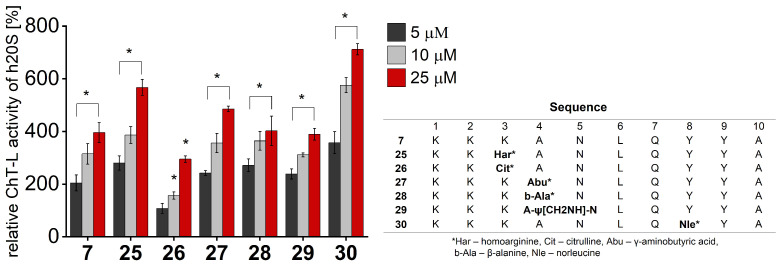
Peptidomimetics based on compound **7** stimulated the ChT-L peptidase of human 20S proteasome. Compound **30** at a 25 µM concentration exhibited a 1.8-fold increase in activity compared to **7**. All activity assays were performed in three independent replicates. Results are expressed as a percentage of activity of the latent 20S and are presented as the mean ± SD. Ordinary one-way ANOVA was used to determine statistical significance (* *p* < 0.05).

When we tested each of the obtained peptidomimetics for their ability to stimulate human 20S proteasome, we found that some of them enhanced ChT-L and C-L peptidases to a higher extent than the parent peptide **7**, with the most remarkable effect for the compound with norleucine introduced at position 8 (**30**), which at a 25 µM concentration, increased the ChT-L activity 1.8 times and the C-L activity 2 times those of the 20S relative to compound **7** (Figure 5 and Figure S7).

**Figure 6:**
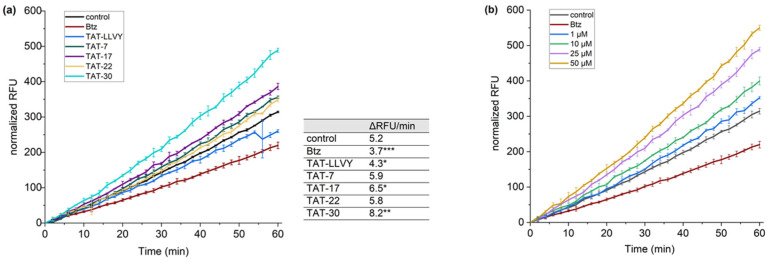
(**a**) The impact of compounds TAT-7, TAT-17, TAT-22, TAT-30, and TAT-LLVY on proteasome-mediated degradation in HEK293T cells, monitored using a TAS3 fluorescent probe. The relative fluorescence units were plotted against time. The selective proteasome inhibitor, bortezomib, used as a negative control, decreased proteasome activity by approximately 30% (*** *p* < 0.001). The most potent activator, TAT-30, was able to increase 20S activity by more than 50% (** *p* < 0.01), and TAT-17 caused a 25% (* *p* < 0.05) gain in activity. The table next to the graph presents the change in fluorescence units per minute for each compound; (**b**) TAT-30 enhanced the proteasome-mediated hydrolysis of the TAS3 probe in a dose-dependent manner. At a 50 µM concentration of TAT-30, the activity of the proteasome increased by about 75%.

The observed gain in the stimulation potency of compounds **17** and **30** and the observed cell permeability of TAT-7 prompted us to include TAT (48–57) and LLVY sequences in compounds **17**, **22**, and **30** and verify if they influence proteasome activity in cells. To achieve this goal, we synthesized and used the recently described cell-permeable TAS3 probe [36]. Model HEK293T cells, treated with the cell-permeable proteasome inhibitor, bortezomib (Btz), were used in these tests as a negative control. Regarding controls, the cells treated with compound **22**, which did not stimulate 20S in in vitro activity assays, were also used, as well as TAT-LLVY-treated cells. To evaluate the propensity of TAT-7, TAT-17, TAT-22, and TAT-30 to enhance proteasome activity, we monitored the fluorescence intensity of the probe over time (Figure 6a). A significant increase in fluorescence intensity compared to the control was observed for the TAT-30 sample. This compound was able to increase proteasome activity by more than 50%. TAT-17 was also able to increase the hydrolysis of the TAS3 probe, but to a lesser extent than TAT-30. In contrast, TAT-7 and TAT-22 had minor impacts on the activity of the proteasome. Subsequently, we also checked how the concentration of TAT-30 affects its ability to stimulate proteasome (Figure 6b). We observed that even 1 µM of TAT-30 facilitated the hydrolysis of the TAS3 substrate, but at higher concentrations, its influence on the proteasome activity increased markedly. Remarkably, even at the highest tested concentration (50 µM), it did not exert a cytotoxic effect on HEK293T cells (Figure S8).

**Figure 7:**
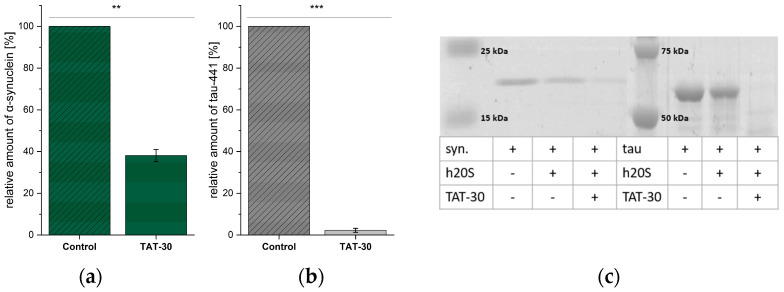
The influence of TAT-30 (10 μM) on the degradation of protein substrates, (**a**) α-synuclein and (**b**) Tau-441, by the human 20S proteasome. Relative quantities of undigested proteins were determined based on the electrophoretic separation of the protein samples after their incubation with the modulator TAT-30 and 20S proteasome or the proteasome alone (control); (**c**) SDS-PAGE electrophoregram with Coomassie Blue-stained bands (representative of three independent replicates). Statistical analysis was performed using one-way ANOVA (** *p* < 0.01, *** *p* < 0.001).

Encouraged by the observed influence of TAT-30 on TAS3 hydrolysis in cells, we decided to test its impact on the degradation of two intrinsically disordered proteins, α-synuclein and Tau-441, by 20S proteasome. These proteins form toxic aggregate species in Parkinson’s and Alzheimer’s disease, respectively, and in their development, the crucial risk factor is age [37,38]. The 20S was incubated with the studied proteins in the absence and presence of the activator. Figure 7 indicates that TAT-30 at a 10 µM concentration enhanced the digestion of α-synuclein about 2.5 times, whereas Tau-441 was almost completely degraded in the presence of the modulator. The obtained results further indicate the potential of TAT-30 to diminish the accumulation of impaired proteins.

## 3. Materials and Methods

### 3.1. General

Fmoc-protected amino acids, N,N’-diisopropylcarbodiimide, Oxyma Pure and trifluoroacetic acid were purchased from Iris Biotech (Marktredwitz, Germany). Cl-MPA ProTide Resin was supplied by CEM (Matthews, NC, USA). Dess-Martin Periodinane was supplied by Fluorochem (Hadfield, UK). Reagents for electrophoresis were purchased from Biorad (Hercules, CA, USA). The cell culture reagents, if not otherwise stated, were purchased from Merck (Darmstadt, Germany). All general purpose solvents (dimethylformamide, dichloromethane, dimethyl sulfoxide) as well as HEPES and Tris were supplied by Fisher Scientific (Waltman, MA, USA). Acetonitrile (Supelco) was purchased from Merck (Darmstadt, Germany).

### 3.2. Peptide/Peptidomimetic Synthesis

All peptides and peptidomimetics were synthesized on a solid support (Cl-MPA ProTide Resin (LL)) using a Liberty Blue microwave peptide synthesizer (CEM, Matthews, NC, USA) and standard Fmoc (9-fluorenylmethoxycarbonyl) chemistry. The coupling of the Fmoc-protected amino acids was carried out utilizing a 1:1 mixture of 0.5 M N,N′-diisopropylcarbodiimide in dimethylformamide (DMF) with 1 M ethyl cyano(hydroxyimino)acetate (Oxyma Pure). To synthesize compound **29** with a reduced peptide bond, Fmoc-Alaninol was oxidized using Dess-Martin Periodinane [39]. The obtained Fmoc-Alaninal was subsequently coupled in a microwave reactor (150 W, 80 °C) to an NLQYYA peptide on a solid support (pre-soaked with acetic acid/diglyme/DMF, 1:5:94 (*v*:*v*:*v*)), followed by a microwave-assisted (150 W, 80 °C) reduction of the formed imine bond with NaBH_3_CN. The following amino acid residues were then coupled using a Liberty Blue synthesizer. Crude peptides/peptidomimetics were purified via RP-HPLC (K2001; Knauer, Berlin, Germany) on a C12 semipreparative Jupiter Proteo column (21.2 mm × 250 mm, 4 μm (Phenomenex, Torrance, CA, USA)) using a linear gradient of acetonitrile in 0.1% aqueous trifluoroacetic acid. The purity of the synthesized compounds was evaluated via analytical RP-HPLC (Varian ProStar 240, Palo Alto, CA, USA) using an XB-C18 Aeris Peptide column (4.6 mm × 150 mm, 3.6 μm, 100 Å, Phenomenex) or a Jupiter Proteo C12 column (4.6 mm × 250 mm, 4 μm, 90 Å, Phenomenex). The identities of pure products were evaluated via ESI-IT-TOF LCMS (Prominence, Shimadzu, Kyoto, Japan) and/or MALDI-TOF MS (autoflex^®^ maX, Bruker, Billerica, MA, USA).

### 3.3. TAMRA Attachment

TAMRA fluorescent tags were attached to the N-terminal α-amino group of the lysine residue of compound **7** and the ε-amino group of the corresponding lysine residue in compound TAT-7 before the final deprotection and cleavage of the compounds from the solid support. To achieve the selective attachment of the label to the proper ε-amino group, the Fmoc-Lys(Mtt)-OH was used in this position during peptide elongation. Mtt protecting group was removed utilizing trifluoroethanol:acetic acid:dichloromethane 2:1:7 (*v*:*v*:*v*) solution. The coupling of 5(6)-carboxytetramethylrhodamine succinimidyl ester (5(6)-TAMRA-SE, Genaxxon Bioscience, Ulm, Germany) was conducted three times. The first coupling was carried out by agitating 2 eq of 5(6)-TAMRA-SE and 6 eq of N,N-diisopropylethylamine (DIEA) with the corresponding peptide resin in DMF for 24 h at room temperature. Two subsequent couplings were performed using 1 eq of 5(6)-TAMRA-SE and 3 eq of DIEA in DMF (2 × 24 h). 

### 3.4. Proteasome Activity Assays

The influence of the obtained peptides/peptidomimetics on the proteasome catalytic activities was tested using human 20S isolated from erythrocytes, and the appropriate fluorogenic substrates (Suc-LLVY-AMC for chymotrypsin-like (ChT-L), Boc-LRR-AMC for trypsin-like (T-L), and Z-LLE-AMC for caspase-like (C-L) activity (Bachem, Bubendorf, Switzerland)). The final concentration of proteasome was 0.001 mg/mL (1.4 nM). Stock solutions of the substrates and the tested peptides/peptidomimetics were prepared in dimethyl sulfoxide (DMSO). The final concentration of the substrates was 100 µM. The compounds were tested in the range of 5 μM to 50 μM. The final concentration of DMSO in the samples was kept constant at 2%. The activity assays were performed in black 96-well plates (COSTAR; Corning, NY, USA) in 50 mM Tris-HCl buffer, pH 8.0, using a 100 μL reaction volume. The release of aminomethylcoumarin (AMC) was measured continuously every 2 min for 60 min, at 37 °C, using Tecan Infinite M200 Pro plate reader (Tecan Trading AG, Männedorf, Switzerland). The excitation and emission wavelengths were set at 380 nm and 460 nm, respectively. The percentage of the substrate hydrolysis in the presence of the activators was calculated in relation to the control (DMSO-treated proteasome) that was regarded as 100%. The influence of all compounds on the chymotrypsin-like activity of human 20S proteasome was studied in three replicates, and on trypsin-like and caspase-like, in two independent replicates. The results are presented as the mean ± SD. Statistical analyses were performed with OriginPro 2021 (OriginLab, Northampton, MA, USA), using ordinary one-way analysis of variance (ANOVA). A *p*-value < 0.05 was considered statistically significant.

### 3.5. Confocal Microscopy

Human embryonic kidney cells (HEK293T, a generous gift of Prof. Grzegorz Węgrzyn, Faculty of Biology, University of Gdańsk, Gdańsk, Poland) were maintained in Dulbecco’s Modified Eagle Medium (DMEM) supplemented with 10% Fetal Bovine Serum (FBS) and penicillin/streptomycin (100 units/mL/100 µg/mL), at 37 °C with 5% CO_2_. Cells were seeded on 24-well plates at a density of 3 × 10^4^ cells per well and incubated in 0.5 mL complete medium for 48 h. Then, the cells were washed with phosphate-buffered saline (PBS), and fresh medium containing peptides labeled with a 5(6)-TAMRA was added to each well at a concentration of 10 µM. After 24 h of incubation, the cells were washed thoroughly with PBS, and a phenol red-free culture medium (FluoroBrite^TM^ DMEM, Gibco; Thermo Fisher Scientific, Waltham, MA, USA) was added. Subsequently, the cells were examined using a Nicon Eclipse TI2 microscope (Nikon Instruments Inc., Melville, NY, USA) and a red filter for TAMRA. The images were merged, and the colocalization of the dye emission was analyzed. 

### 3.6. Determination of the Sites of Compound **7** Cleavage by 20S Proteasome

Peptide **7** (250 μM) was incubated at 37 °C in the presence of latent human 20S proteasome (0.0125 mg/mL) in 25 mM Tris-HCl buffer, pH 8.0. The reaction volume was 100 μL. The reaction was stopped after 3 h through the addition of 2.5 μL of 10% TFA. The identities of the peptide bonds digested by the proteasome were determined based on *m*/*z* signals detected via ESI-IT-TOF LCMS (Prominence, Shimadzu, Kyoto, Japan).

### 3.7. The Effect of Peptides/Peptidomimetics on the 20S Activity in Cellulo (TAS3 Assay) 

A TAS3 probe was synthesized according to the procedure described in [36]. HEK293T cells were seeded in black 96-well plates at 7 × 10^3^ cells/well and incubated in DMEM supplemented with 10% FBS and penicillin/streptomycin (100 units/mL/100 µg/mL) at 37 °C with 5% CO_2_. After 48 h, the cells were left in complete medium or preincubated with 10 µM bortezomib. After this time, the cells were treated with the tested peptides/peptidomimetics (1–50 µM in medium). Each sample was prepared in triplicate. The plates were incubated for an additional 30 min. Subsequently, the medium was removed, and 100 µL of 10 µM TAS3 in Krebs-Ringer Bicarbonate (KRBH) buffer, containing the tested modulator, was added to the wells. The fluorescence intensity was monitored every 2 min over a 90 min period at 37 °C using a Tecan Infinite M200 Pro plate reader (Tecan Trading AG, Männedorf, Switzerland) with the excitation and emission wavelengths set at 485 nm and 535 nm, respectively. A linear regression analysis was performed for the last 60 min of the measurement. Any increase or decrease in activity of the 20S was compared to that of the control (cells incubated with TAS3 probe in KRBH buffer) and calculated based on the change in fluorescence units per time [40]. To determine statistical significance, ordinary one-way ANOVA was used.

### 3.8. MTT Assay

The cytotoxicity of the tested peptides/peptidomimetics was estimated using the MTT assay. HEK293T cells were seeded in 96-well plates (7 × 10^3^ cells per well) and incubated in 0.1 mL complete medium for 2 days at 37 °C with 5% CO_2_. Next, growth medium was substituted with medium supplemented with the indicated concentrations of the tested compounds. The cells were incubated for 24 h, and then 5 mg/mL MTT (3-[4,5-dimethylthiazol-2-yl]-2,5-diphenyltetrazolium bromide (Acros Organics, Geel, Belgium) solution in PBS buffer was added to a final concentration of 0.5 mg/mL. The plates were incubated for 4 h at 37 °C, and then the solution was replaced with 150 µL of solubilizing agent (DMSO). After the complete solubilization, purple formazan, produced as a result of MTT reduction by metabolically active cells, was quantified by measuring the absorbance at 570 nm with a reference filter of 690 nm.

### 3.9. Protein Substrate Degradation Assay

α-synuclein (rPeptide, Watkinsville, GA, USA) and Tau-441 (Novus Biologicals, Centennial, CO, USA) were dissolved in 20 mM HEPES, pH 7.4, to 0.5 mg/mL. The samples were prepared by mixing human proteasome 20S (final concentration—1 pmol), the protein (387 pmol of α-synuclein or 61 pmol of Tau-441), and DMSO (control) or the modulator TAT-30 (10 µmol) dissolved in DMSO. The total sample volume was 10 μL. The final concentration of the organic solvent never exceeded 0.05%. The samples were incubated at 37 °C for 1.5 h. The reaction was stopped with 4 × Laemmli buffer, and then heated at 75 °C for 10 min. The results were analyzed electrophoretically after loading 8 μL of each sample onto a 10% (for Tau-441) or 12% (for synuclein) SDS–PAGE gel (Figure S9). The amounts of undigested proteins were calculated from the gels stained with Coomassie Blue-based reagent, InstantBlue^TM^. A quantitative image analysis was carried out with GS-800 Calibrated Densitometer and the Quantity One^®^ 1-D analysis software v. 4.6.9 provided by the manufacturer (Bio-Rad, Hercules, CA, USA). The band intensities of a protein incubated with the 20S and a modulator were compared with the intensity of the band corresponding to the protein incubated with the 20S alone, which was considered to be 100%. Each result represents an average of the data from three experiments and is presented as the mean ± SD. One-way ANOVA was used to determine statistical significance.

## 4. Conclusions

In this work, we designed and synthesized a series of peptides and peptidomimetics based on the C-terminus of the Rpt5 subunit of the natural proteasome regulator, 19S, and verified their capacity to stimulate the activity of human 20S proteasome. At a concentration of 25 µM, compound **30** with norleucine introduced at position 8, facilitated the turnover of small fluorogenic ChT-L and C-L substrates by the 20S by about 7 and 8.5 times, respectively, compared to the vehicle control. We also proved that the activators, after the attachment of the known cell-penetrating peptide TAT (48–57), can penetrate model noncancerous cells, HEK293T. Studies performed with the use of a cell-permeable substrate of the proteasome, TAS3, indicated, for the first time, the ability of peptides/peptidomimetics to stimulate proteasome activity in cells. Further work with the use of senescent cells will determine whether the best compound, TAT-30, can counteract the decline in proteasome activity observed during aging. In the presented study, we also showed that TAT-30 was capable of significantly increasing the proteasome propensity to degrade aggregation-prone proteins α-synuclein and Tau-441.

Collectively, the results presented here show that proteasomal stimulation by TAT-30 and other peptides/peptidomimetics derived from the binding regions of the natural proteasome regulators may be promising leads for compensating for the unbalanced proteostasis found in aging and age-related diseases. 

## Data Availability

Data presented in this study are available upon request from the corresponding authors.

## Supplementary Materials

The following supporting information can be downloaded at https://www.mdpi.com/article/10.3390/ijms25094663/s1.
