# Supplementary material for: Rpt5-Derived Analogs Stimulate Human Proteasome Activity in Cells and Degrade Proteins Forming Toxic Aggregates in Age-Related Diseases
Source: Int J Mol Sci. 2024 Apr 25;25(9):4663. doi: 10.3390/ijms25094663 (PMC11082943; doi:10.3390/ijms25094663)
Supplement: Supplementary file 1 [file ijms-25-04663-s001.zip › ijms-2947822-supplementary.pdf]

Supplementary figures

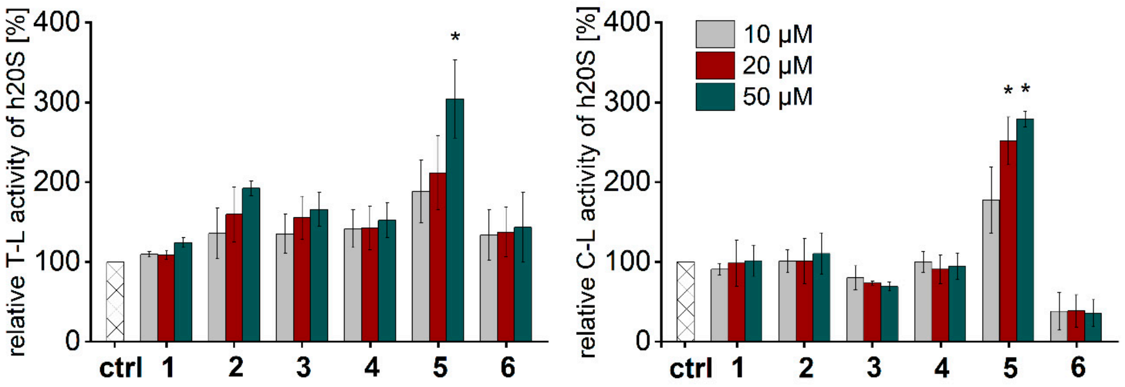

Figure S1. The capacity of compounds **1**-**6** for stimulating T-L and C-L activities of human 20S proteasome. Only the peptide corresponding to the *C*-terminus of the Rpt5 subunit (**5**) increased the activities of the 20S in a dose-dependent manner. All activity assays were performed in two independent replicates. Results are expressed as a percentage of activity of the latent 20S and are presented as the mean ± SD. Ordinary one-way ANOVA was used to determine statistical significance ($^{*}p < 0.05$).

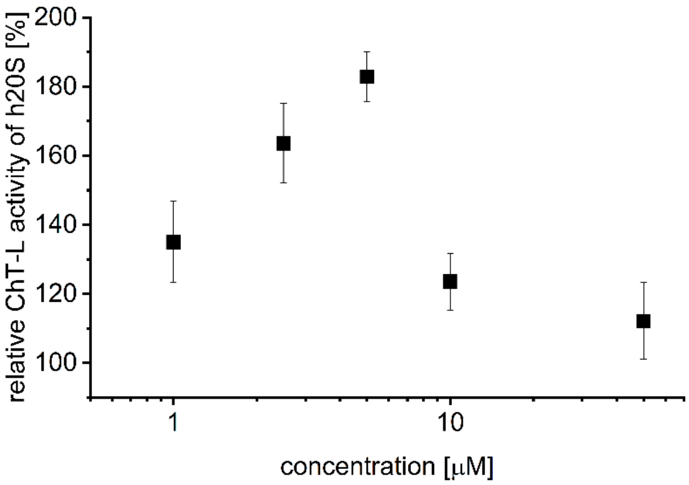

Figure S2. The chymotrypsin-like activity of human 20S proteasome was activated by Rpt6-derived *C*-terminal peptide (compound **6**) at lower concentrations with maximum activation observed at 5 uM, which was followed by a systematic decrease in efficacy. The results of three independent replicates are presented as the mean ± SD.

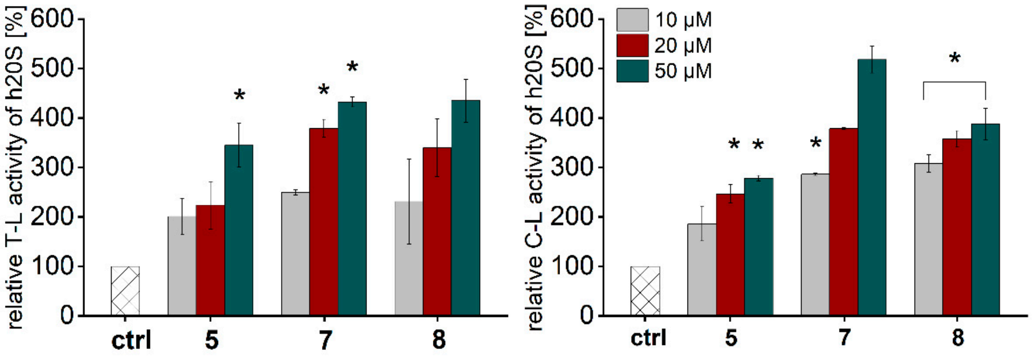

Figure S3. The influence of the peptide chain length on T-L and C-L activity of the human 20S proteasome. The activity assays were performed in two independent replicates. Results are expressed as a percentage of activity of the latent 20S and are presented as the mean ± SD. Ordinary one-way ANOVA was used to determine statistical significance ($^{*}p < 0.05$).

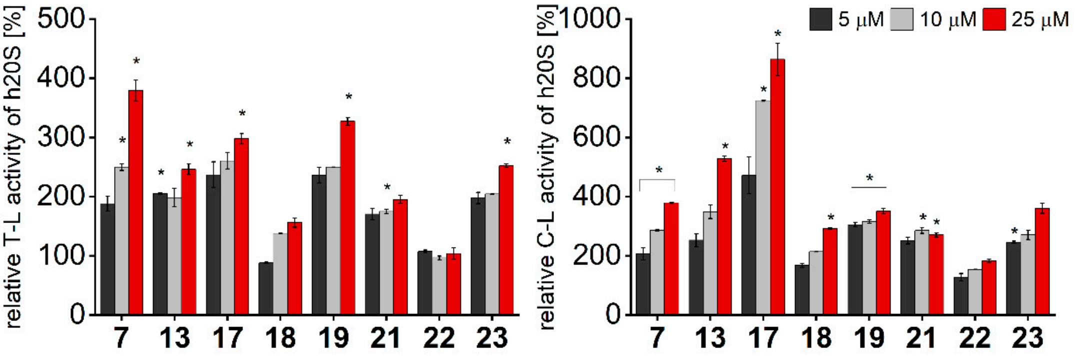

Figure S4. The influence of compound **7** analogs on the T-L and C-L activity of human 20S proteasome. The results of two independent replicates are presented as the mean ± SD. Ordinary one-way ANOVA was used to determine statistical significance ($^{*}p < 0.05$).

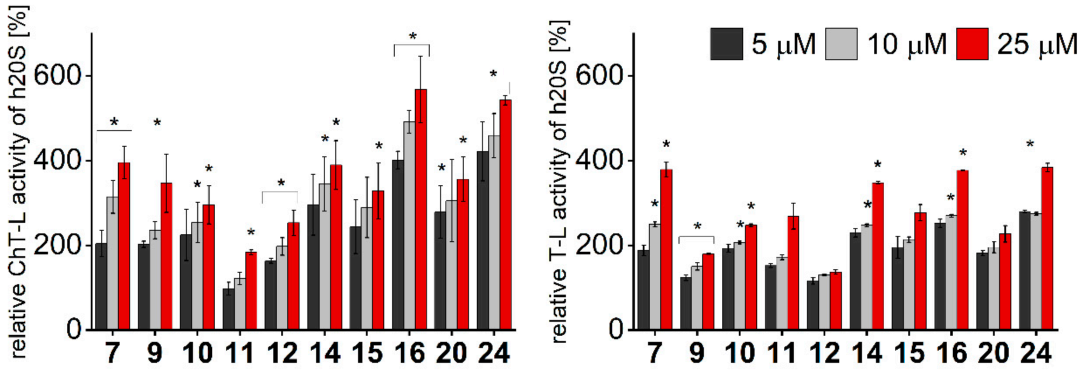

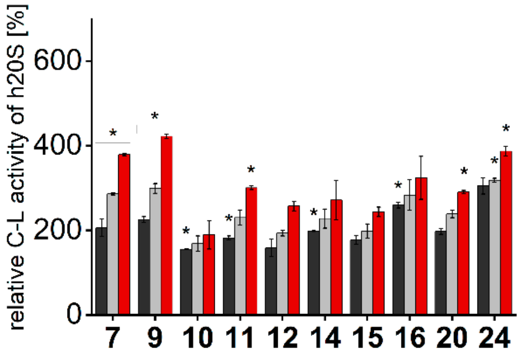

Sequence

	1	2	3	4	5	6	7	8	9	10
**7**	K	K	K	A	N	L	Q	Y	Y	A
**9**	**Q**	K	K	A	N	L	Q	Y	Y	A
**10**	**E**	K	K	A	N	L	Q	Y	Y	A
**11**	K	**Q**	K	A	N	L	Q	Y	Y	A
**12**	K	**E**	K	A	N	L	Q	Y	Y	A
**14**	K	K	**Q**	A	N	L	Q	Y	Y	A
**15**	K	K	**E**	A	N	L	Q	Y	Y	A
**16**	K	K	K	**N**	N	L	Q	Y	Y	A
**20**	K	K	K	A	**D**	L	Q	Y	Y	A
**24**	K	K	K	A	N	L	**D**	Y	Y	A

Figure S5. The influence of the compound **7** analogs on the ChT-L, T-L, and C-L activity of human 20S proteasome. For ChT-L activity three, and for T-L and C-L activity two independent replicates were performed. The results are presented as mean ± SD. Ordinary one-way ANOVA was used to determine statistical significance ($^{*}p < 0.05$).

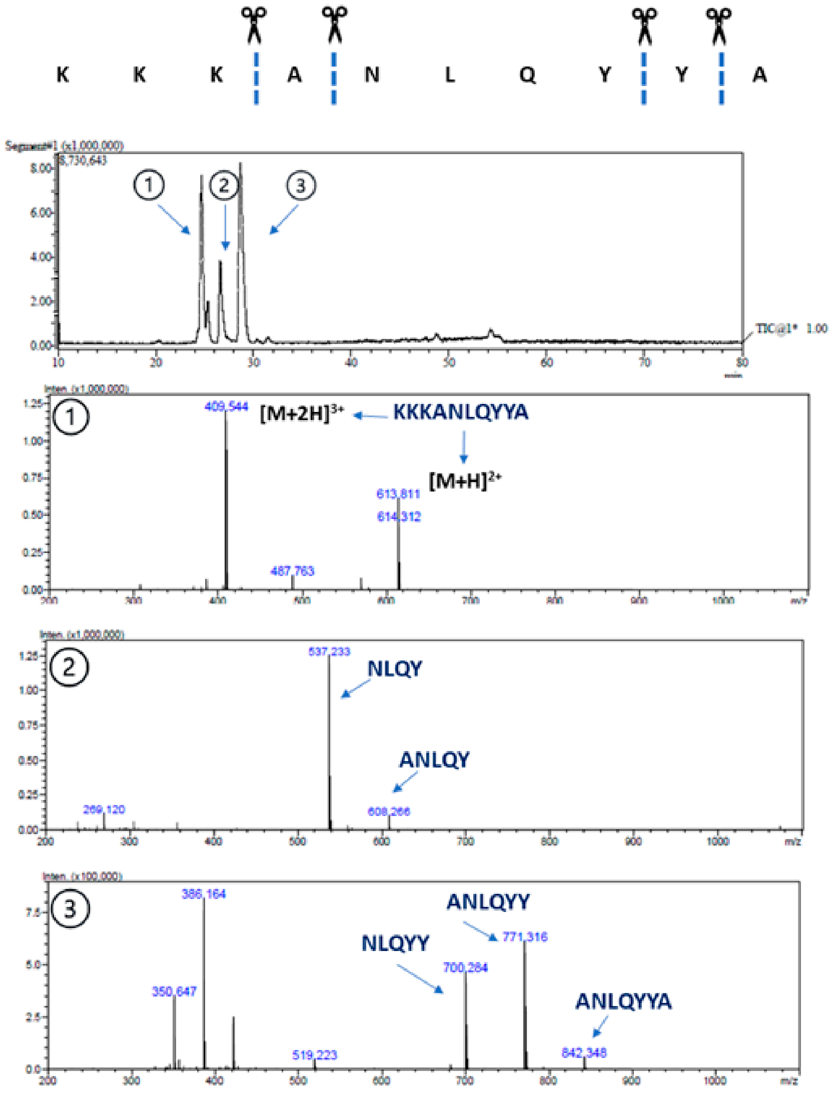

Figure S6. Cleavage sites of the peptide **7** by the 20S proteasome determined based on *m*/*z* signals corresponding to the individual fragments of the compound, detected by ESI-IT-TOF LCMS (Prominence, Shimadzu).

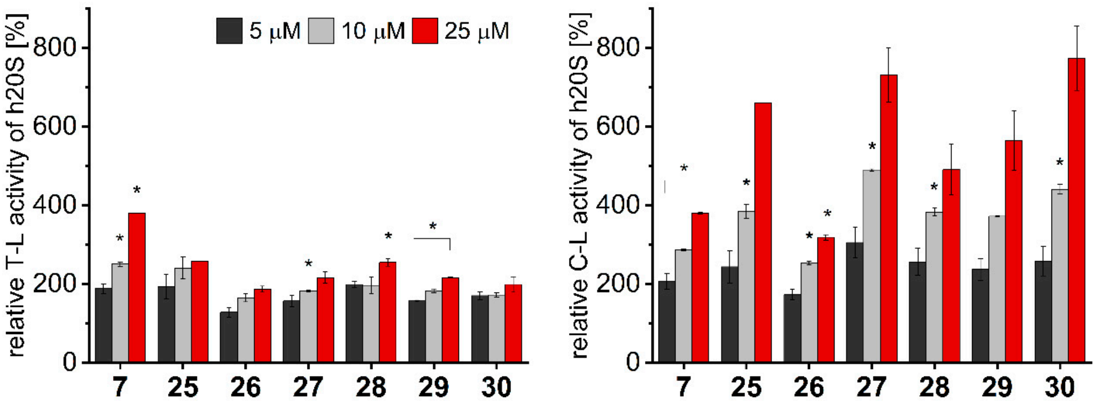

Figure S7. The influence of peptidomimetics based on compound **7** on the T-L and C-L activity of human 20S proteasome. The results of two independent replicates are presented as the mean ± SD. Ordinary one-way ANOVA was used to determine statistical significance ($^{*}p < 0.05$).

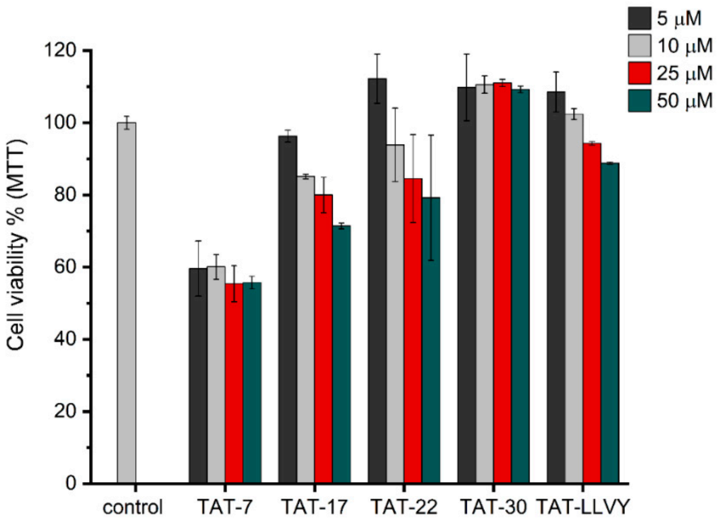

Figure S8. The viability of HEK293T cells was not significantly affected after treatment with **TAT-7**, **TAT-17**, **TAT-22**, and **TAT-LLVY. TAT-30** did not exert any cytotoxic effect on of the cells. The results of the MTT assay, conducted in three independent replicates, are presented as the mean ± SD.

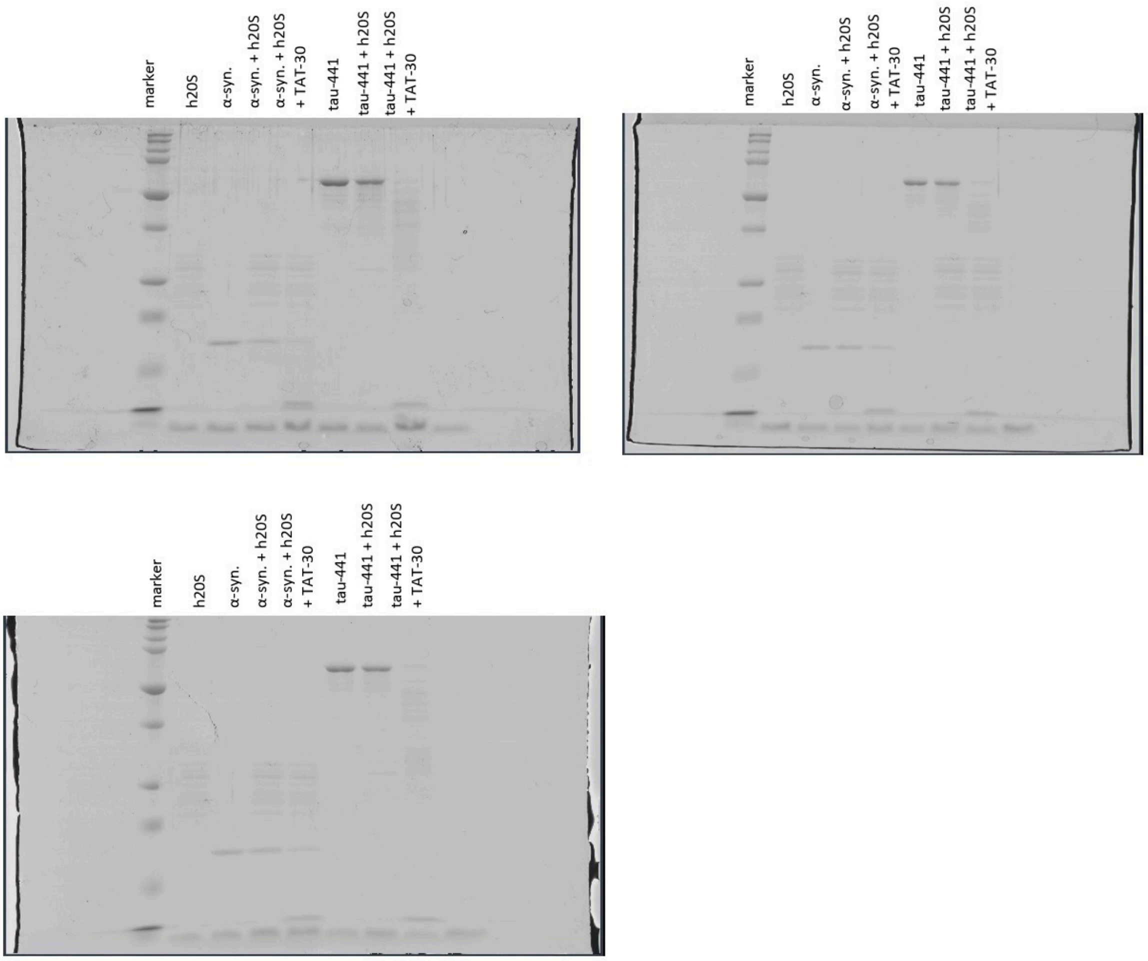

Figure S9. SDS PAGE gels of α-synuclein and Tau-441 degradation by the 20S in the presence of **TAT-30** activator at 10 μM concentration. Precision Plus Protein™ Dual Xtra Prestained Protein Standards (Biorad) was used as a marker (bands: 2, 5, 10, 15, 20, 25, 37, 50, 75, 100, 150, and 250kDa).
